# Wave Dispersion Analysis of Fluid Conveying Nanocomposite Shell Reinforced by MWCNTs Considering the Effect of Waviness and Agglomeration Efficiency

**DOI:** 10.3390/polym13010153

**Published:** 2021-01-01

**Authors:** Mohammad Alkhedher, Pouyan Talebizadehsardari, Arameh Eyvazian, Afrasyab Khan, Naeim Farouk

**Affiliations:** 1 Mechanical Engineering Department, Abu Dhabi University, Abu Dhabi 59911, UAE; mohammad.alkhedher@adu.ac.ae; 2 Metamaterials for Mechanical, Biomechanical and Multiphysical Applications Research Group, Ton Duc Thang University, Ho Chi Minh City 758307, Vietnam; ptsardari@tdtu.edu.vn; 3 Faculty of Applied Sciences, Ton Duc Thang University, Ho Chi Minh City 758307, Vietnam; 4 Institute of Research and Development, Duy Tan University, Da Nang 550000, Vietnam; 5 Faculty of Electrical—Electronic Engineering, Duy Tan University, Da Nang 550000, Vietnam; 6 Institute of Engineering and Technology, Department of Hydraulics and Hydraulic and Pneumatic Systems, South Ural State University, Lenin Prospect 76, 454080 Chelyabinsk, Russia; khana@susu.ru; 7 Mechanical Engineering Department, College of Engineering, Prince Sattam bin Abdulaziz University, Alkharj 16273, Saudi Arabia; n.mohammed@psau.edu.sa

**Keywords:** wave dispersion analysis, MWCNT-reinforced nanocomposite, first-order shear deformable shell theory, waviness factor, random orientation factor, agglomeration factor, fluid flow

## Abstract

The current paper is aimed to investigate the effects of waviness, random orientation, and agglomeration factor of nanoreinforcements on wave propagation in fluid-conveying multi-walled carbon nanotubes (MWCNTs)-reinforced nanocomposite cylindrical shell based on first-order shear deformable theory (FSDT). The effective mechanical properties of the nanocomposite cylindrical shell are estimated employing a combination of a novel form of Halpin-Tsai homogenization model and rule of mixture. Utilized fluid flow obeys Newtonian fluid law and it is axially symmetric and laminar flow and it is considered to be fully developed. The effect of flow velocity is explored by implementing Navier-Stokes equation. The kinetic relations of nanocomposite shell are calculated via FSDT. Moreover, the governing equations are derived using the Hamiltonian approach. Afterward, a method which solves problems analytically is applied to solve the obtained governing equations. Effects of a wide range of variants such as volume fraction of MWCNTs, radius to thickness ratio, flow velocity, waviness factor, random orientation factor, and agglomeration factor on the phase velocity and wave frequency of a fluid conveying MWCNTs-reinforced nanocomposite cylindrical shell were comparatively illustrated and the results were discussed in detail.

## 1. Introduction 

One substantial concern of engineers in designing structures and researchers in analyzing structures is the durability and permanence of the structure while it is subjected to various kinds of static and dynamic loadings. This issue has become an interesting topic on manufacturing, designing and analyzing structures made of various materials. Composite materials owing to their superb chemical and physical properties are one of suitable candidate to be selected as constituent materials of structures [1]. Composite materials are a mixture of at least two kind of materials with different physical and chemical properties. Generally, there are two main categories of constituent materials: reinforcement and matrix. Matrix surrounds the reinforcements and the reinforcements enhance the matrix properties by imparting their mechanical and physical properties. These reinforcements have been applied to strengthen the properties of their base materials for a myriad of engineering applications such as automotive, marine, aerospace and aircraft structures, biomedical media, and sports goods. Another advantage is the wide choice of materials which can be used as matrix and reinforcements, for instance, polymer, ceramic, and metal materials can be used as the matrix. Owing to these great capabilities, composite structures have received wide attention of researchers and engineers to analyze mechanical behavior like buckling, vibration and wave propagation of such structure. Kant and Swaminathan [2] investigated vibrational behavior of simply supported sandwich composite plates within the framework of refined higher-order shear deformable theory (HSDT). HSDT was applied by Tripathi, et al. [3] to investigate vibrational behaviors of laminated composite conical shells with consideration of randomness sensitivity. Thai and Kim [4] studied the natural frequency of laminated plates in the framework of two unknowns refined plate theory using the Navier solution technique. The nonlinear vibrational response of laminated plates on the basis of nonlinear Von Kármán’s theory was surveyed by Houmat [5] using the hierarchical finite element method (FEM). Verma [6] probed the wave propagation behavior of a desired direction in laminated composite plates. Panda and Singh [7] also solved the nonlinear thermal vibration problem of post-buckled laminated composite spherical panel embedded with shape memory alloy fiber using nonlinear FEM based on HSDT. Dynamic analysis of the laminated composite shells on the basis of the higher-order zigzag theory by a 2D FEM was performed by Kumar, et al. [8]. Nedri, et al. [9] analyzed the natural frequency of cross-ply laminated composite plates lying on an elastic medium via a refined hyperbolic shear deformation theory. Free vibrational and stability behavior of laminated composite flat and curved panels under thermomechanical loading were examined by Panda and Katariya [10]. Moreover, the incremental harmonic balance method was employed by Dey and Ramachandra [11] to evaluate the nonlinear transverse dynamic problem of laminated composite cylindrical shells under static axial partial loading and periodic radial point loading in the framework of Von Kármán’s nonlinear shell theory. Barouni and Saravanos [12] explored guided wave propagation characteristics of infinite laminated composite plates based on a layerwise semi-analytical theory. The frequency oscillation of various shapes of shell with concentrated and cutouts mass made of laminated composite according to third-order shear deformable theory (TSDT) was analyzed by Chaubey, et al. [13]. Recently, Gao, et al. [14] investigated the guided wave dispersion behavior of anisotropic composite laminates utilizing the Legendre polynomials and state-vector formalism. Nastos and Saravanos [15] probed the guided wave propagation of a sandwich plate based on Daubechies wavelet functions in conjunction with a layerwise laminate plate theory. Recently, Safaei [16] studied natural frequency oscillation of an embedded laminated composite plates with consideration of porosity effect. 

Besides, the reinforcements can be in various scales and shapes including fiber as macro-reinforcement and particle as nano-reinforcement. The materials reinforced with the nanoreinforcements are called nanocomposite. Carbon-base materials such as carbon fiber, graphene, graphene oxide (GO), graphene platelets (GPLs), and carbon nanotubes (CNTs) are among the best candidates for being used as reinforcement. CNTs can be divided to Single-walled CNT (SWCNT) and Double-walled CNT (DWCNT) and Multi-walled CNT (MWCNT). They have similar properties and application but, among them, MWCNTs have slightly better properties. The existence of multi-walled nanotube makes MWCNTs more appropriate to utilize as reinforcements. Several investigations have addressed the mechanical responses of reinforced nanocomposite structures [17,18,19,20,21]. For example, Heshmati and Yas [22] investigated forced and free frequency response of non-uniform beams made of MWCNTs-strengthened nanocomposite based on Timoshenko beam theory utilizing FEM. Kiani [23] discussed the vibrational response of CNTs-reinforced nanocomposite spherical panels within the framework of FSDT. Based on HSDT, Wang, et al. [24] analyzed the static and dynamic characteristics of the nanocomposite doubly-curved shallow shells reinforced with GPLs. Free vibration and buckling analyses of pre-stressed cylindrical GPL- reinforced shells (with nonuniform distribution) were performed by Liu, et al. [25]. Kumar and Srinivas [26] used Navier’s solution technique to solve the transient vibrational problem of MWCNTs-reinforced nanocomposite plate resting on Pasternak substrate based on TSDT. Moreover, the dispersion of flexural waves in GPLs-reinforced nanocomposite cylindrical shells with respect to porosity was surveyed by Ebrahimi, et al. [27] according to FSDT. Propagation of elastic waves in composite plates reinforced with CNTs based upon FSDT was explored by Karami, et al. [28]. Frequency analysis of GPLs-reinforced multi-layer nanocomposite beams lying on a viscoelastic foundation through HSDT was presented by Qaderi, et al. [29] using Navier’s solution technique. Ghassabi, et al. [30] performed wave propagation modeling of doubly curved thick shells made of CNT-reinforced composite based on three-dimensional theory. Barati and Zenkour [31] investigated the dynamic oscillation of imperfect nanocomposite shells reinforced by graphene platelets using Galerkin’s method. In this work, various patterns have been considered for porosity and GPLs distributions. Recently, wave propagation and buckling characteristics of thermally excited nanocomposite plate reinforced with GO powder resting on an elastic foundation was evaluated through refined HSDT by Ebrahimi, et al. [32], Ebrahimi, et al. [33]. Lal and Markad [34] examined the postbuckling behavior of MWCNTs-reinforced laminated nanocomposite beam resting on an elastic medium under thermomechanical loading. Lately, Lee [35] probed the buckling and nonlinear transient problems of hybrid nanocomposite cylindrical panels with or without delamination about a cutout utilizing Hewitt and Malherbe model.

Several problems still challenge the development of the reinforced composite structures. Orientation, waviness, and agglomeration of reinforcements are some of these problems which can substantially affect the mechanical behavior of composite structures. In this context, the analysis and design of composite structures concerning the mentioned factors are of crucial significance. Therefore, the influences of reinforcements’ orientation, waviness, and aggregation on the mechanical response of composite structures have been surveyed by several researchers. Jam, et al. [36] explored the natural frequency of CNTs-reinforced composite cylindrical panels considering the aspect ratio and waviness effects of nanofillers using a three-dimensional elasticity theory. Vibration oscillations of the Timoshenko beam reinforced with randomly oriented CNTs were assessed by Rashidifar and Ahmadi [37] utilizing FEM. The dynamic response of the composites reinforced with long randomly oriented fiber was evaluated by Sepahvand [38] based on FSDT. Kamarian, et al. [39] probed the influence of CNTs’ aggregation on dynamic analysis of CNTs-reinforced nanocomposite conical shells via FSDT using generalized differential quadrature method. Furthermore, Tahouneh [40] investigated the impact of CNTs’ waviness and aspect ratio on the free vibration analysis of embedded CNT-reinforced nanocomposite annular plates in the framework of FSDT and HSDT. Both waviness and aggregation influences of the nanoreinforcements on free vibrational behavior of CNTs-reinforced nanocomposite skew plate were examined by García-Macías and Castro-Triguero [41]. Recently, characteristics of propagation of waves in a multiscale hybrid nanocomposite beams, plates and shells regarding the influence of CNTs agglomeration were analyzed in [42,43,44].

Analysis of propagation of waves in structures is one of significant analyses that study mechanical behavior of structures. Besides, this analysis can be helpful in designing, modeling and analyzing structure which are utilized in different systems. Also, in non-destructive tests and structural health monitoring, this analysis can be advantageous [45,46,47]. It is quite sufficient to motivate us to perform wave propagation analysis of structures especially reinforced composite structures. The literature review, however, showed no study addressing wave propagation analysis of fluid conveying MWCNTs-reinforced nanocomposite cylindrical shell in terms of MWCNTs orientation, waviness, and agglomeration within the framework of a novel form of Halpin-Tsai micromechanical model. Indeed, the orientation, waviness, and agglomeration of reinforcements affect remarkably mechanical response of reinforced composite structures. Also, these influences have not been examined on responses of propagated wave in MWCNTs-reinforced nanocomposite structures. One of important application of selected structure (cylindrical shell) is in pipelines and pressure vessels. In another word, composite pipes has been established in flow and gathering lines, tanks and distribution systems associated with natural gas transmission. Thus, the present paper is aimed at filling this gap.

In the present work, the effects of MWCNTs’ orientation, waviness, and agglomeration on wave dispersion behavior of a fluid conveying MWCNT-reinforced polymeric nanocomposite were investigated in the framework of a novel form of Halpin-Tsai homogenization model. In the new form of Halpin-Tsai micromechanical model, the noted effects can be covered and studied implicitly. Plus, FSDT was implemented to attain kinetic relations of nanocomposite cylindrical shells. In the next step, the obtained governing equations were analytically solved to probe the effect of various variables on the variation of phase velocity and wave frequency of fluid conveying MWCNTs-reinforced nanocomposite cylindrical shells.

## 2. Theory and Formulation 

**Figure 1:**
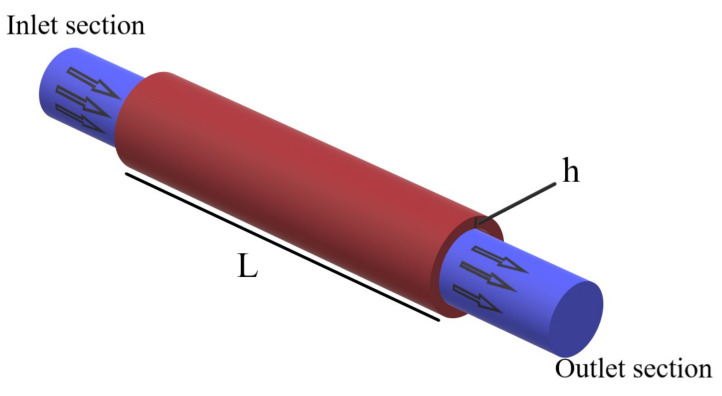
Schematic of fluid conveying MWCNT-reinforced nanocomposite shell.

A schematic view of a fluid-conveying MWCNTs-strengthened cylindrical shell with respective length and height of *L* and *h* is depicted in Figure 1. MWCNTs are distributed into the polymer matrix.

### 2.1. Homogenization Procedure

In the current section, a new form of Halpin-Tsai homogenization model and rule of the mixture was applied to determine the material properties of the nanocomposite shell. The effective mechanical properties of nanocomposite shell will be calculated as [48]:

(1) $$E(z)=E_{m}\frac{1+2\eta \zeta V_{r}}{1-\zeta V_{r}},\;\;\;\eta =\frac{L_{r}}{d_{r}},\;\;\;\zeta =\frac{\frac{E_{r}}{E_{m}}-1}{\frac{E_{r}}{E_{m}}+2\eta }$$

(2) $$\rho (z)=\rho_{m}V_{m}+\rho_{r}V_{r}$$

(3) $$\upsilon (z)=\upsilon_{m}V_{m}+\upsilon_{r}V_{r}$$

in which *E* represent Young’s modulus, *ρ* represent mass density*,* and *υ* represent Poisson’s ratio. Furthermore, subscripts of *r* and *m* stand for MWCNT and polymer matrix, respectively. *L_r_* is MWCNTs’ length and *d_r_* shows its diameter. *V_m_* and *V_r_* denote volume fraction of matrix and MWCNTs, respectively which have the following relationship:

(4) $$V_{m}+V_{r}=1$$

**Figure 2:**
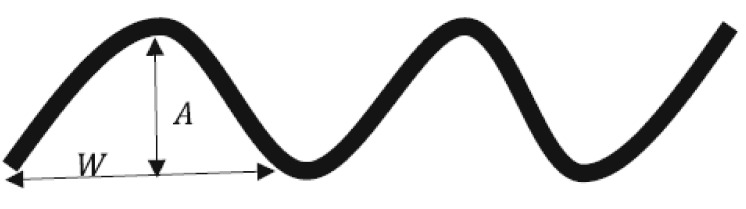
The schematic model of the wavy MWCNT [48].

Waviness, random orientation, and agglomeration factors can significantly influence the value of elastic modulus. The mentioned factors can be considered through the following equation [48]:

(5) $$\;\zeta =\frac{\left(\frac{f_{R}f_{W}f_{A}E_{c}}{E_{m}}-1\right)}{\left(\frac{f_{R}f_{W}f_{A}E_{c}}{E_{m}}+2\eta \right)}a$$

in which *f_R_* is the random orientation factor. MWCNTs were assumed to be oriented randomly in three directions in this research and the value of random orientation factor equals 1/6 [49]. If MWCNTs are considered to be randomly oriented in two dimensions, *f_R_*=1/3. *f_W_* is waviness factor which can be defined as [48]:

(6) $$f_{W}=1-\frac{A}{W}$$

in which *A* stands for amplitude a wavy MWCNT and *W* stands for the amplitude and half-wavelength of a wavy MWCNT, as indicated in Figure 2. In the present investigation, the waviness factor was assumed to be 0.6. 

*f_A_* is agglomeration factor which has the following definition [48]:

(7) $$f_{A}=e^{-\alpha {\left(V_{r}\right)}^{\lambda }}$$

where α and λ are referred to the degree of MWCNTs agglomeration which were assumed to be 10 and 0.9, respectively.

### 2.2. Kinetic Relations 

Pursuant to the first-order shear deformable shell theory, the displacement fields at every point of a fluid-conveying MWCNTs-reinforced nanocomposite cylindrical shell can be defined as [43]:

(8) $$u_{1}(x,\psi ,z,t)=u(x,\psi ,t)+z\theta_{x}(x,\psi ,t)$$

(9) $$u_{2}(x,\psi ,z,t)=v(x,\psi ,t)+z\theta_{\psi }(x,\psi ,t)$$

(10) $$u_{3}(x,\psi ,z,t)=w(x,\psi ,t)$$

where *u* is axial displacement, *v* is circumferential displacement, *w* is lateral displacements*, θ_x_* is rotation about axial direction*_,_* and *θ_ψ_* is rotation about circumferential direction respectively. Hence, the strains of a cylindrical shell that are not zero can be written as:

(11) $$\varepsilon_{xx}=\frac{\partial u}{\partial x}+z\frac{\partial \theta_{x}}{\partial x}$$

(12) $$\varepsilon_{\psi \psi }=\frac{1}{R}\left(w+\frac{\partial v}{\partial \psi }+z\frac{\partial \theta_{\psi }}{\partial \psi }\right)$$

(13) $$\varepsilon_{xz}=\theta_{x}+\frac{\partial w}{\partial x}$$

(14) $$\varepsilon_{x\psi }=\frac{\partial v}{\partial x}+\frac{1}{R}\frac{\partial u}{\partial \psi }+\frac{z}{R}\frac{\partial \theta_{x}}{\partial \psi }+z\frac{\partial \theta_{\psi }}{\partial x}$$

(15) $$\varepsilon_{\psi z}=\theta_{\psi }+\frac{1}{R}\frac{\partial w}{\partial \psi }-\frac{v}{R}$$

in which *R* denotes radius of cylindrical shell. Then, to achieve Euler-Lagrange equations of MWCNTs-reinforced cylindrical shell, Hamiltonian approach was applied and it can be stated as:

(16) $$\int_{t_{0}}^{t_{1}}\left[\delta U-\delta K-\delta W_{nc}\right]dt=0$$

in which *U* indicates strain energy, *K* indicates kinetic energy*_,_* and *W_nc_* indicates work done by external loadings. The strain energy’s variation for an elastic structure is written as follows:

(17) $$\begin{array}{cc} \delta U & =\int_{-\frac{h}{2}}^{\frac{h}{2}}\int_{0}^{2\pi }\int_{0}^{L}\sigma_{ij}\delta \varepsilon_{ij}Rdxd\phi dz\\ & \;=\int_{-\frac{h}{2}}^{\frac{h}{2}}\int_{0}^{2\pi }\int_{0}^{L}\left[\sigma_{xx}\delta \varepsilon_{xx}+\sigma_{\psi \psi }\delta \varepsilon_{\psi \psi }+\sigma_{xz}\delta \varepsilon_{xz}+\sigma_{\psi z}\delta \varepsilon_{\psi z}+\sigma_{x\psi }\delta \varepsilon_{x\psi }\right]Rdxd\phi dz\\ & \;=\int_{-\frac{h}{2}}^{\frac{h}{2}}\int_{0}^{2\pi }\int_{0}^{L}\left[\begin{array}{c} \sigma_{xx}\delta \left(\frac{\partial u}{\partial x}+z\frac{\partial \theta_{x}}{\partial x}\right)+\sigma_{\psi \psi }\delta \left(\frac{1}{R}\left(w+\frac{\partial v}{\partial \psi }+z\frac{\partial \theta_{\psi }}{\partial \psi }\right)\right)\\ +\sigma_{xz}\delta \left(\theta_{x}+\frac{\partial w}{\partial x}\right)++\sigma_{\psi z}\delta \left(\theta_{\psi }+\frac{1}{R}\frac{\partial w}{\partial \psi }-\frac{v}{R}\right)\\ \sigma_{x\psi }\delta \left(\frac{\partial v}{\partial x}+\frac{1}{R}\frac{\partial u}{\partial \psi }+\frac{z}{R}\frac{\partial \theta_{x}}{\partial \psi }+z\frac{\partial \theta_{\psi }}{\partial x}\right) \end{array}\right]Rdxd\psi dz \end{array}$$

The resultant forces and momentum can be stated as follow:

(18) $$\left\{\begin{array}{l} N_{xx}\\ N_{x\psi }\\ N_{\psi \psi } \end{array}\right\}=\int_{-\frac{h}{2}}^{\frac{h}{2}}\left\{\begin{array}{l} \sigma_{xx}\\ \sigma_{x\psi }\\ \sigma_{\psi \psi } \end{array}\right\}dz$$

(19) $$\left\{\begin{array}{l} M_{xx}\\ M_{x\psi }\\ M_{\psi \psi } \end{array}\right\}=\int_{-\frac{h}{2}}^{\frac{h}{2}}\left\{\begin{array}{l} \sigma_{xx}\\ \sigma_{x\psi }\\ \sigma_{\psi \psi } \end{array}\right\}zdz$$

(20) $$\left\{\begin{array}{l} Q_{xz}\\ Q_{z\psi } \end{array}\right\}=\kappa_{s}\int_{-\frac{h}{2}}^{\frac{h}{2}}\left\{\begin{array}{l} \sigma_{xz}\\ \sigma_{z\psi } \end{array}\right\}dz$$

By using Equations (18)–(20), Equation (17) can be rewritten in the following form:

(21) $$\delta U=\int_{-\frac{h}{2}}^{\frac{h}{2}}\int_{0}^{2\pi }\int_{0}^{L}\left[\begin{array}{l} N_{xx}\frac{\partial \delta u}{\partial x}+M_{xx}\frac{\partial \delta \theta_{x}}{\partial x}+\frac{N_{\psi \psi }}{R}\left(\frac{\partial \delta v}{\partial \psi }+\delta w\right)+\frac{M_{\psi \psi }}{R}\frac{\partial \delta \theta_{\psi }}{\partial x}\\ N_{x\psi }\left(\frac{1}{R}\frac{\partial \delta u}{\partial \psi }+\frac{\partial \delta v}{\partial x}\right)+M_{x\psi }\left(\frac{1}{R}\frac{\partial \delta \theta_{x}}{\partial \psi }+\frac{\partial \delta \theta_{\psi }}{\partial x}\right)\\ +Q_{xz}\left(\frac{\partial \delta w}{\partial x}+\delta \theta_{x}\right)+Q_{z\psi }\left(\delta \theta_{\psi }+\frac{1}{R}\frac{\partial \delta w}{\partial \psi }-\frac{1}{R}\delta v\right) \end{array}\right]Rdxd\psi dz$$

Moreover, the variation of kinetic energy can be described by:

(22) $$\delta K=\int_{-\frac{h}{2}}^{\frac{h}{2}}\int_{0}^{2\pi }\int_{0}^{L}\rho (z)\left[{\left(\delta {\dot{u}}_{x}\right)}^{2}+{\left(\delta {\dot{u}}_{\psi }\right)}^{2}+{\left(\delta {\dot{u}}_{z}\right)}^{2}\right]Rdxd\psi dz$$

Dot-subscript is related to differentiation with respect to time. 

Herein, the viscous fluid flow in MWCNTs-reinforced cylindrical shells is supposed to be, Newtonian, laminar, fully developed and axially symmetric [50]. Therefore, the Navier-Stokes equation will be used. The equation of momentum of fluid flow can be stated by:

(23) $$-\frac{\partial P}{\partial R}+\frac{\partial \tau_{Rx}}{\partial x}-\frac{\tau_{\psi \psi }}{R}+\frac{1}{R}\frac{\partial \tau_{R\psi }}{\partial \psi }=\rho_{f}\frac{dV_{R}}{dt}$$

in above equation *ρ_f_* and *P* stand for density of the fluid and pressure of the fluid. Due to the reciprocating identity in the contact points between the speed and acceleration of the structure and fluid, the following equations are developed:

(24) $$V_{R}=\frac{dw}{dt}$$

(25) $$\frac{d}{dt}=\frac{\partial }{\partial t}+v_{x}\frac{\partial }{\partial x}$$

in which *v*_x_ stands for the mean velocity of fluid flow; shear stress (*τ*) and viscosity (*μ_f_*) relations are formulated as:

(26) $$\tau_{R\psi }=\frac{\mu_{f}}{R}\frac{\partial V_{R}}{\partial \psi }$$

(27) $$\tau_{\psi \psi }=2\mu_{f}\frac{V_{R}}{R}$$

(28) $$\tau_{Rx}=R\tau_{R\psi }$$

In the above equation the parameters *μ_f_* and *V_R_* are the viscosity and fluid flow of the fluid, respectively.

The variation of work done by the exterior loading can be stated as:

(29) $$\delta \Pi_{W}=\int_{-\frac{h}{2}}^{\frac{h}{2}}\int_{0}^{2\pi }\int_{0}^{L}\left(\frac{\mu_{f}}{R^{3}}\frac{\partial^{2}V_{R}}{\partial \psi^{2}}+\mu_{f}\frac{\partial^{2}V_{R}}{\partial x^{2}}-\frac{2\mu_{f}}{R^{2}}V_{R}-\rho_{f}\frac{d^{2}w}{dt^{2}}\right)\delta wRdxd\psi dz$$

Therefore, to attain the motion equations of the cylindrical shell, Equations (21), (22) and (29) will be replaced into Equation (16) leading to the following relations:

(30) $$\frac{\partial N_{xx}}{\partial x}+\frac{1}{R}\frac{\partial N_{x\psi }}{\partial \psi }=I_{0}\frac{\partial^{2}u}{\partial t^{2}}+I_{1}\frac{\partial^{2}\theta_{x}}{\partial t^{2}}$$

(31) $$\frac{\partial N_{x\psi }}{\partial x}+\frac{1}{R}\frac{\partial N_{\psi \psi }}{\partial \psi }+\frac{Q_{z\psi }}{R}=I_{0}\frac{\partial^{2}v}{\partial t^{2}}+I_{1}\frac{\partial^{2}\theta_{\psi }}{\partial t^{2}}$$

(32) $$\begin{array}{l} \frac{\partial Q_{xz}}{\partial x}+\frac{1}{R}\frac{\partial Q_{z\psi }}{\partial \psi }-\frac{N_{\psi \psi }}{R}-\rho_{f}h_{f}v_{x}^{2}\frac{\partial^{2}w}{\partial x^{2}}+\mu_{f}h_{f}v_{x}\left[\frac{\partial^{3}w}{\partial x^{3}}+\frac{1}{R}\left(\frac{\partial^{3}w}{\partial x\partial \psi^{2}}-2\frac{\partial w}{\partial x}\right)\right]\\ =I_{0}\frac{\partial^{2}w}{\partial t^{2}}+\rho_{f}h_{f}\left[\frac{\partial^{2}w}{\partial t^{2}}+2v_{x}\frac{\partial^{2}w}{\partial x\partial t}\right]+\mu_{f}h_{f}\left[\frac{\partial^{3}w}{\partial t\partial x^{2}}+\frac{1}{R^{2}}\left(\frac{\partial^{3}w}{\partial t\partial \psi^{2}}-2\frac{\partial w}{\partial t}\right)\right] \end{array}$$

(33) $$\frac{\partial M_{xx}}{\partial x}+\frac{1}{R}\frac{\partial M_{x\psi }}{\partial \psi }-Q_{xz}=I_{1}\frac{\partial^{2}u}{\partial t^{2}}+I_{2}\frac{\partial^{2}\theta_{x}}{\partial t^{2}}$$

(34) $$\frac{\partial M_{x\psi }}{\partial x}+\frac{1}{R}\frac{\partial M_{\psi \psi }}{\partial \psi }-Q_{\psi z}=I_{1}\frac{\partial^{2}v}{\partial t^{2}}+I_{2}\frac{\partial^{2}\theta_{\psi }}{\partial t^{2}}$$

where

(35) $$\left\{\begin{array}{l} I_{0}\\ I_{1}\\ I_{2} \end{array}\right\}=\int_{-\frac{h}{2}}^{\frac{h}{2}}\left\{\begin{array}{l} 1\\ z\\ z^{2} \end{array}\right\}\rho (z)dz$$

in which *κ_s_* is a factor for shear correction which is taken as 5/6.

Integrating the aforementioned equations over the thickness of shell, the following relations will be obtained:

(36) $$N_{xx}=A_{11}\frac{\partial u}{\partial x}+B_{11}\frac{\partial \theta_{x}}{\partial x}+\frac{A_{12}}{R}\left(\frac{\partial v}{\partial \psi }+w\right)+\frac{B_{12}}{R}\frac{\partial \theta_{\psi }}{\partial \psi }$$

(37) $$N_{x\psi }=A_{66}\left(\frac{1}{R}\frac{\partial u}{\partial \psi }+\frac{\partial v}{\partial x}\right)+B_{66}\left(\frac{1}{R}\frac{\partial \theta_{x}}{\partial \psi }+\frac{\partial \theta_{\psi }}{\partial x}\right)$$

(38) $$N_{\psi \psi }=A_{12}\frac{\partial u}{\partial x}+B_{12}\frac{\partial \theta_{x}}{\partial x}+\frac{A_{11}}{R}\left(\frac{\partial v}{\partial \psi }+w\right)+\frac{B_{11}}{R}\frac{\partial \theta_{\psi }}{\partial \psi }$$

(39) $$M_{xx}=B_{11}\frac{\partial u}{\partial x}+D_{11}\frac{\partial \theta_{x}}{\partial x}+\frac{B_{12}}{R}\left(\frac{\partial v}{\partial \psi }+w\right)+\frac{D_{12}}{R}\frac{\partial \theta_{\psi }}{\partial \psi }$$

(40) $$M_{x\psi }=B_{66}\left(\frac{1}{R}\frac{\partial u}{\partial \psi }+\frac{\partial v}{\partial x}\right)+D_{66}\left(\frac{1}{R}\frac{\partial \theta_{x}}{\partial \psi }+\frac{\partial \theta_{\psi }}{\partial x}\right)$$

(41) $$M_{\psi \psi }=B_{12}\frac{\partial u}{\partial x}+D_{12}\frac{\partial \theta_{x}}{\partial x}+\frac{B_{11}}{R}\left(\frac{\partial v}{\partial \psi }+w\right)+\frac{D_{11}}{R}\frac{\partial \theta_{\psi }}{\partial \psi }$$

(42) $$Q_{xz}=A_{55}^{s}\left(\frac{\partial w}{\partial x}+\theta_{x}\right)$$

(43) $$Q_{\psi z}=A_{55}^{s}\left(-\frac{v}{R}+\frac{1}{R}\frac{\partial w}{\partial \psi }+\theta_{\psi }\right)$$

in which

(44) $$\left[\begin{array}{ccc} A_{11} & B_{11} & D_{11}\\ A_{12} & B_{12} & D_{12}\\ A_{66} & B_{66} & D_{66} \end{array}\right]=\int_{-\frac{h}{2}}^{\frac{h}{2}}\left[\begin{array}{c} Q_{11}\\ Q_{12}\\ Q_{66} \end{array}\right]\left[\begin{array}{ccc} 1 & z & z^{2} \end{array}\right]dz$$

(45) $$\;\;A_{55}^{s}=\kappa_{s}\int_{-\frac{h}{2}}^{\frac{h}{2}}Q_{66}dz$$

where $Q_{11}=\frac{E}{1-\upsilon^{2}}\;,\;Q_{12}=Q_{11}\upsilon \;,\;Q_{66}=Q_{11}\frac{1-\upsilon }{2}$. 

At last, by mixing Equations (21)–(27) with Equation (31), the governing ss of fluid conveying MWCNT-reinforced nanocomposite cylindrical shells can be obtained as written below:

(46) $$\begin{array}{l} A_{11}\frac{\partial^{2}u}{\partial x^{2}}+B_{11}\frac{\partial^{2}\theta_{x}}{\partial x^{2}}+\frac{A_{12}}{R}\left(\frac{\partial^{2}v}{\partial x\partial \psi }+\frac{\partial w}{\partial x}\right)\\ +\frac{B_{12}}{R}\frac{\partial^{2}\theta_{\psi }}{\partial x\partial \psi }+\frac{A_{66}}{R}\left(\frac{1}{R}\frac{\partial^{2}u}{\partial \psi^{2}}+\frac{\partial^{2}v}{\partial x\partial \psi }\right)\\ +\frac{B_{66}}{R}\left(\frac{1}{R}\frac{\partial^{2}\theta_{x}}{\partial \psi^{2}}+\frac{\partial^{2}\theta_{\psi }}{\partial x\partial \psi }\right)-I_{0}\frac{\partial^{2}u}{\partial t^{2}}-I_{1}\frac{\partial^{2}\theta_{x}}{\partial t^{2}}=0 \end{array}$$

(47) $$\begin{array}{l} A_{66}\left(\frac{1}{R}\frac{\partial^{2}u}{\partial x\partial \psi }+\frac{\partial^{2}v}{\partial x^{2}}\right)+B_{66}\left(\frac{1}{R}\frac{\partial^{2}\theta_{x}}{\partial x\partial \psi }+\frac{\partial^{2}\theta_{\psi }}{\partial x^{2}}\right)+\frac{A_{12}}{R}\frac{\partial^{2}u}{\partial x\partial \psi }\\ +\frac{B_{12}}{R}\frac{\partial^{2}\theta_{x}}{\partial x\partial \psi }+\frac{A_{11}}{R^{2}}\left(\frac{\partial^{2}v}{\partial \psi^{2}}+\frac{\partial w}{\partial \psi }\right)\\ +\frac{B_{11}}{R^{2}}\frac{\partial^{2}\theta_{\psi }}{\partial \psi^{2}}+\frac{A_{55}^{s}}{R}\left(\theta_{\psi }+\frac{1}{R}\frac{\partial w}{\partial \psi }-\frac{v}{R}\right)-I_{0}\frac{\partial^{2}v}{\partial t^{2}}-I_{1}\frac{\partial^{2}\theta_{\psi }}{\partial t^{2}}=0 \end{array}$$

(48) $$\begin{array}{cc} \; & A_{55}^{s}\left(\frac{\partial \theta_{x}}{\partial x}+\frac{\partial^{2}w}{\partial x^{2}}\right)+\frac{A_{55}^{s}}{R}\left(\frac{\partial \theta_{\psi }}{\partial \psi }+\frac{1}{R}\frac{\partial^{2}w}{\partial \psi^{2}}-\frac{1}{R}\frac{\partial v}{\partial \psi }\right)-\frac{A_{12}}{R}\frac{\partial u}{\partial x}-\frac{B_{12}}{R}\frac{\partial \theta_{x}}{\partial x}-\frac{A_{11}}{R^{2}}\left(\frac{\partial v}{\partial \psi }+w\right)\\ \; & -\frac{B_{11}}{R^{2}}\frac{\partial \theta_{\psi }}{\partial \psi }-\rho_{f}h_{f}v_{x}^{2}\frac{\partial^{2}w}{\partial x^{2}}+\mu_{f}h_{f}v_{x}\left[\frac{\partial^{3}w}{\partial x^{3}}+\frac{1}{R}\left(\frac{\partial^{3}w}{\partial x\partial \psi^{2}}-2\frac{\partial w}{\partial x}\right)\right]-I_{0}\frac{\partial^{2}w}{\partial t^{2}}\\ \; & -\rho_{f}h_{f}\left[\frac{\partial^{2}w}{\partial t^{2}}+2v_{x}\frac{\partial^{2}w}{\partial x\partial t}\right]-\mu_{f}h_{f}\left[\frac{\partial^{3}w}{\partial t\partial x^{2}}+\frac{1}{R^{2}}\left(\frac{\partial^{3}w}{\partial t\partial \psi^{2}}-2\frac{\partial w}{\partial t}\right)\right]=0 \end{array}$$

(49) $$\begin{array}{l} B_{11}\frac{\partial^{2}u}{\partial x^{2}}+D_{11}\frac{\partial^{2}\theta_{x}}{\partial x^{2}}+\frac{B_{12}}{R}\left(\frac{\partial^{2}v}{\partial x\partial \psi }+\frac{\partial w}{\partial x}\right)+\frac{D_{12}}{R}\frac{\partial^{2}\theta_{\psi }}{\partial x\partial \psi }\\ +\frac{B_{66}}{R}\left(\frac{1}{R}\frac{\partial^{2}u}{\partial \psi^{2}}+\frac{\partial^{2}v}{\partial x\partial \psi }\right)+\frac{D_{66}}{R}\left(\frac{1}{R}\frac{\partial^{2}\theta_{x}}{\partial \psi^{2}}+\frac{\partial^{2}\theta_{\psi }}{\partial x\partial \psi }\right)\\ -A_{55}^{s}\left(\theta_{x}+\frac{\partial w}{\partial x}\right)-I_{1}\frac{\partial^{2}u}{\partial t^{2}}-I_{2}\frac{\partial^{2}\theta_{x}}{\partial t^{2}}=0 \end{array}$$

(50) $$\begin{array}{l} B_{66}\left(\frac{1}{R}\frac{\partial^{2}u}{\partial x\partial \psi }+\frac{\partial^{2}v}{\partial x^{2}}\right)+D_{66}\left(\frac{1}{R}\frac{\partial^{2}\theta_{x}}{\partial x\partial \psi }+\frac{\partial^{2}\theta_{\psi }}{\partial x^{2}}\right)+\frac{B_{12}}{R}\frac{\partial^{2}u}{\partial x\partial \psi }\\ +\frac{D_{12}}{R}\frac{\partial^{2}\theta_{x}}{\partial x\partial \psi }+\frac{B_{11}}{R^{2}}\left(\frac{\partial^{2}v}{\partial \psi^{2}}+\frac{\partial w}{\partial \psi }\right)+\frac{D_{11}}{R^{2}}\frac{\partial^{2}\theta_{\psi }}{\partial \psi^{2}}\\ -A_{55}^{s}\left(\theta_{\psi }+\frac{1}{R}\frac{\partial w}{\partial \psi }-\frac{v}{R}\right)-I_{1}\frac{\partial^{2}v}{\partial t^{2}}-I_{2}\frac{\partial^{2}\theta_{\psi }}{\partial t^{2}}=0 \end{array}$$

## 3. Analytical Solution Scheme 

A method that consist of an exponential function was considered to analytically solve the obtained governing equations of fluid-conveying MWCNT-reinforced nanocomposite cylindrical shells. Therefore, the displacement fields will be:

(51) $$\left\{\begin{array}{l} u\\ v\\ w\\ \theta_{x}\\ \theta_{\psi } \end{array}\right\}=\left\{\begin{array}{l} U_{m}\\ V_{m}\\ W_{m}\\ \Theta_{xm}\\ \Theta_{\psi m} \end{array}\right\}e^{\left[i(k_{x}x+k_{n}\psi -\omega_{m}t)\right]}$$

in Equation (51) the displacement amplitudes are representing by *U_m_*, *V_m_,* and *W_m_*, while rotation amplitudes are representing by Θ*_xm_* and Θ*_ψm_*. Moreover, *k_x_* is longitudinal wave number and *k_n_* is circumferential wave number. Finally, *ω_m_* shows the circular natural frequency. By inserting displacement fields from Equation (51) in Equations (46)–(50), the following relation can be achieved:

(52) $$\left(\left[\begin{array}{ccccc} K_{11}+iC_{11}\omega_{m}-M_{11}\omega_{m}^{2} & K_{12}+iC_{12}\omega_{m}-M_{12}\omega_{m}^{2} & K_{13}+iC_{13}\omega_{m}-M_{13}\omega_{m}^{2} & K_{14}+iC_{14}\omega_{m}-M_{14}\omega_{m}^{2} & K_{15}+iC_{15}\omega_{m}-M_{15}\omega_{m}^{2}\\ K_{21}+iC_{21}\omega_{m}-M_{21}\omega_{m}^{2} & K_{22}+iC_{22}\omega_{m}-M_{22}\omega_{m}^{2} & K_{23}+iC_{23}\omega_{m}-M_{23}\omega_{m}^{2} & K_{24}+iC_{24}\omega_{m}-M_{24}\omega_{m}^{2} & K_{25}+iC_{25}\omega_{m}-M_{25}\omega_{m}^{2}\\ K_{31}+iC_{31}\omega_{m}-M_{31}\omega_{m}^{2} & K_{32}+iC_{32}\omega_{m}-M_{32}\omega_{m}^{2} & K_{33}+iC_{33}\omega_{m}-M_{33}\omega_{m}^{2} & K_{34}+iC_{34}\omega_{m}-M_{34}\omega_{m}^{2} & K_{35}+iC_{35}\omega_{m}-M_{35}\omega_{m}^{2}\\ K_{41}+iC_{41}\omega_{m}-M_{41}\omega_{m}^{2} & K_{42}+iC_{42}\omega_{m}-M_{42}\omega_{m}^{2} & K_{34}+iC_{34}\omega_{m}-M_{34}\omega_{m}^{2} & K_{44}+iC_{44}\omega_{m}-M_{44}\omega_{m}^{2} & K_{45}+iC_{45}\omega_{m}-M_{45}\omega_{m}^{2}\\ K_{51}+iC_{51}\omega_{m}-M_{51}\omega_{m}^{2} & K_{52}+iC_{52}\omega_{m}-M_{52}\omega_{m}^{2} & K_{35}+iC_{35}\omega_{m}-M_{35}\omega_{m}^{2} & K_{54}+iC_{54}\omega_{m}-M_{54}\omega_{m}^{2} & K_{55}+iC_{55}\omega_{m}-M_{55}\omega_{m}^{2} \end{array}\right]\right)\left[\begin{array}{cc} \; & U_{m}\\ \; & V_{m}\\ \; & W_{m}\\ \; & \Theta_{xm}\\ \; & \Theta_{\psi m} \end{array}\right]=0$$

By solving the following relation, natural frequency can be calculated:

(53) $$\left|{\left[K+iC\omega_{m}-M\omega_{m}^{2}\right]}_{5\times 5}\right|=0$$

where *K, C* and *M* illustrates stiffness, damping and mass matrices, respectively. The elements of every matrix are provided in the Appendix A.

By dividing circular frequency to 2π, the wave frequency can be computed. Furthermore, by setting $k_{x}=k_{n}=\beta$, the phase velocity can be computed through:

(54) $$c_{p}=\frac{\omega_{m}}{\beta }$$

## 4. Numerical Results and Discussion

In present section, several plots are provided to clarify the influence of different parameters on propagation of wave in fluid-conveying MWCNTs-reinforced nanocomposite cylindrical shells. In this research, the thickness of the shell was supposed to be 5 cm; also, both the length and radius of the shell were taken 30 times greater than its thickness. The material properties of MWCNTs and polystyrene are given in Table 1. Moreover, the random orientation factor (1/6) was taken into account for all diagrams. First of all, the introduced methodology was validated by evaluating the obtained outcomes with those stated by Pradhan, et al. [51], Wang and Wu [52], and Li, et al. [53]. The obtained non-dimensional natural frequencies of an isotropic shell ($\bar{\omega }=R\omega \sqrt{\frac{\rho \left(1-\upsilon^{2}\right)}{E}}$) were compared to results of other investigations for Clamp-Clamp boundary condition. Based on Table 2, it exists a good agreement between the results of present modeling and results of compared studies.

**Figure 3:**
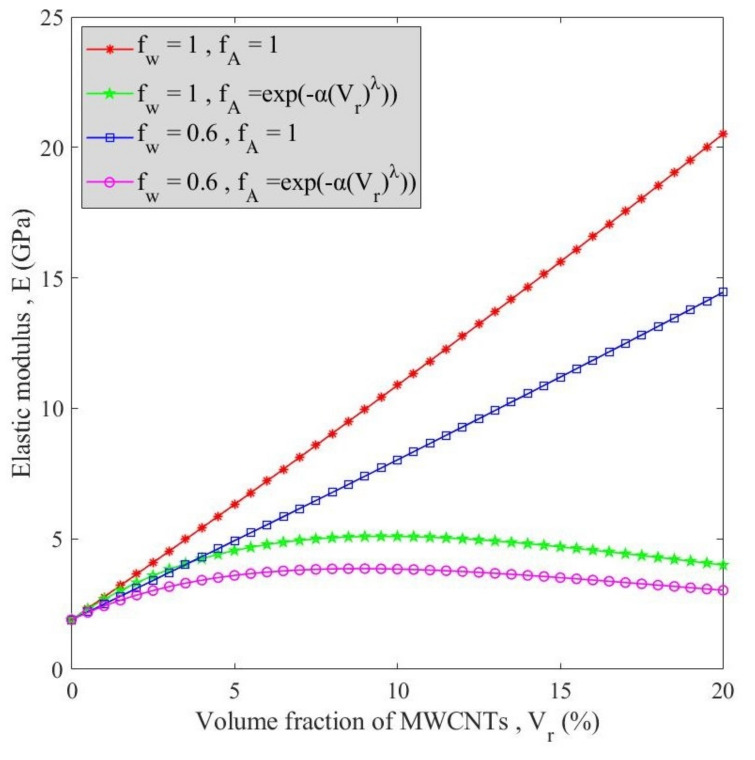
The influence of waviness and agglomeration factors on variation of elastic modulus of MWCNT-reinforced nanocomposite versus MWCNTs’ volume fraction.

Figure 3 illustrates the effect of agglomeration and waviness factors on the changes of elastic modulus versus volume fraction of MWCNTs. As observed, regardless of the agglomeration and waviness factor, the elastic modulus linearly increased with an increment of volume fraction of MWCNTs. However, by involving agglomeration and waviness factor, the trend changed such that by increasing the MWCNTs content, elastic modulus increases up to a peak followed by a decline. Typically, incrementing the volume fraction of MWCNTs makes the structure stiffer; but at a certain amount of MWCNTs, agglomeration of MWCNTs negatively affects the stiffness of the structure. Moreover, this diagram suggests the higher influence of the agglomeration factor (rather than waviness) on elastic modulus.

**Figure 4:**
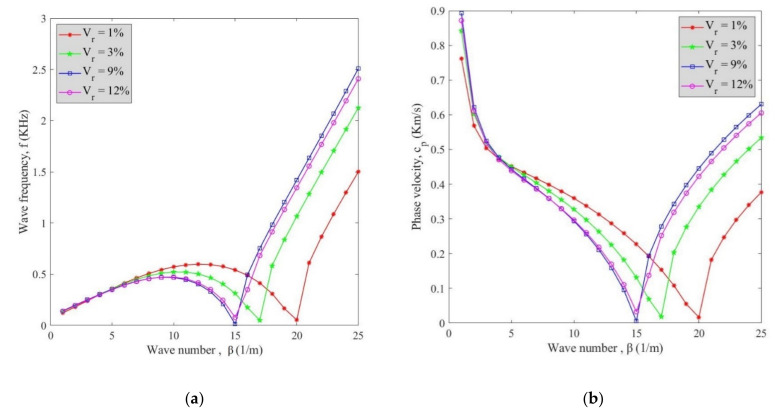
Variation of (**a**) wave frequency and (**b**) phase velocity versus wave number for various volume fraction of MWCNTs.

Variation of wave frequency (**a**) and phase velocity (**b**) against wave number for various MWCNTs’ volume fractions is illustrated in Figure 4 considering all factors and *v_x_* = 2000. It can be said that both phase velocity and wave frequency increased by an increment in the volume fraction of MWCNTs but the curve of *V_r_* = 12% had lower values than *V_r_* = 9% which can be assigned to the agglomeration factor. Moreover, because of the existence of fluid in the cylindrical shell, there is a damping effect which caused a reduction in the phase velocity and wave frequency at a certain wave number after which, the phase velocity, and wave frequency rose. The wave number at which the damping effect was observed increased by selecting lower volume fractions of MWCNTs. Generally, by rising the wave number, the wave frequency first increases followed by a decline at damping wave number; after that, it again rises. For the phase velocity, a reduction is first observed until a damping wave number which is then followed by an increase.

**Figure 5:**
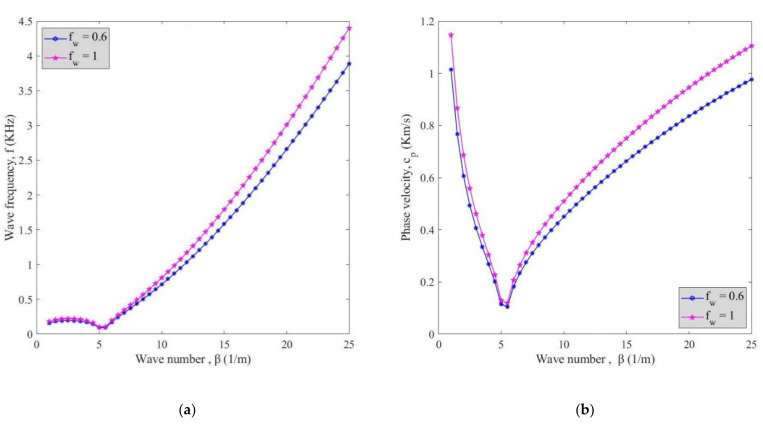
The effect of waviness factor on changes of (**a**) wave frequency and (**b**) phase velocity against wave number.

The effect of waviness factor on the changes of wave frequency (**a**) and phase velocity (**b**) against wave number is surveyed in Figure 5 at *V_r_* = 5%. To clarify the effect of waviness factor on wave frequency and phase velocity, agglomeration factor, and fluid flow velocity were not taken into account. The waviness factor descended the variation of phase velocity and wave frequency. Moreover, by rising the wave number, phase velocity first declined and then grew after damping wave number (*β* = 6).

**Figure 6:**
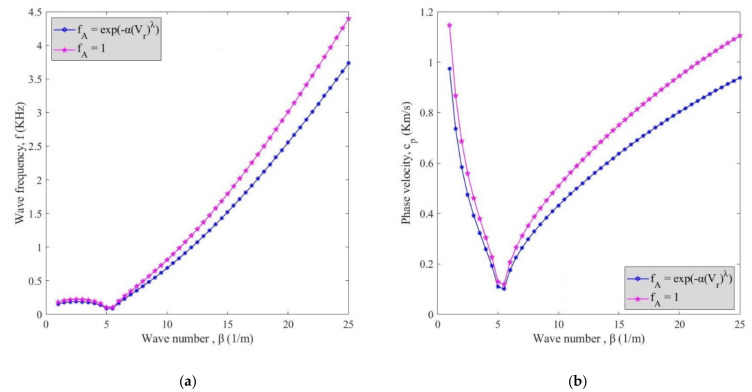
The effect of agglomeration factor on changes of (**a**) wave frequency and (**b**) phase velocity against wave number.

Figure 6 depicts the influence of agglomeration on the changes of wave frequency (**a**) and phase velocity (**b**) by wave number alternation regardless of waviness factor and fluid flow velocity at *V_r_* = 5%. As mentioned before, similar to waviness, the agglomeration had a negative and decreasing effect. Hence, the trend of diagrams (Figure 6) is similar to that of waviness (Figure 5). In another word, phase velocity and wave frequency of the curve are higher when agglomeration was neglected as compared with the case considering the agglomeration effect. A comparison of this figure with the previous one shows that the curve with the agglomeration factor exhibited higher values than the one considering the waviness factor indicating the higher significance of the agglomeration factor rather than waviness which should be considered in the design and analysis of reinforced structures.

**Figure 7:**
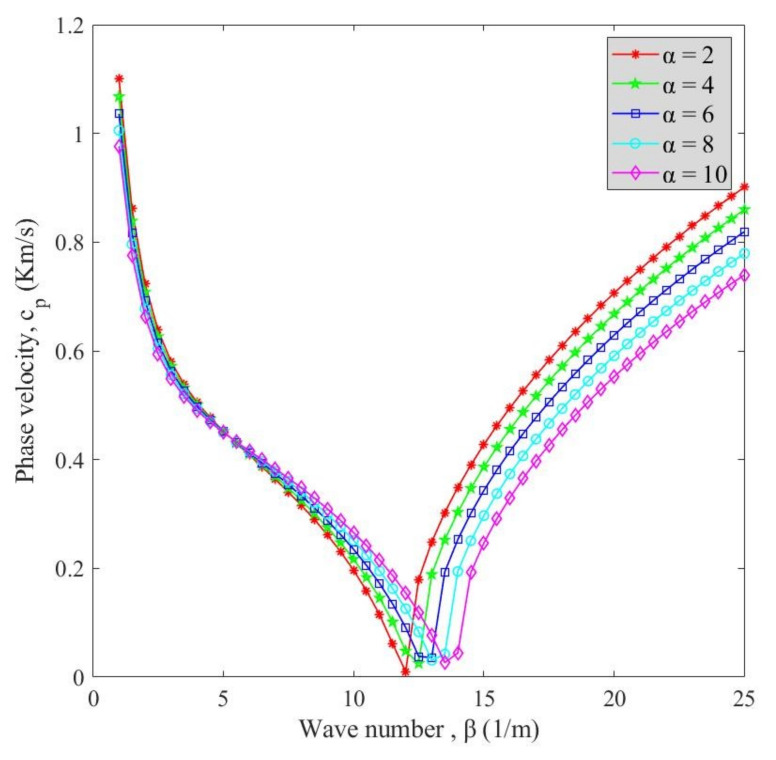
Variation of phase velocity versus wave number for different agglomeration degrees (α).

Variation of phase velocity with the wave number is demonstrated in Figure 7 for various agglomeration degrees (α) at *v_x_* = 2000 and *V_r_* = 5%. Noteworthy, the growth of parameter α exhibited a greater influence of the MWCNT aggregated state on the response of nanocomposite; thus, phase velocity values are diminished by choosing greater values for parameter α. Also, the damping effect occurred at higher wave numbers in cases with higher α parameter values. Thereupon, wiping out the agglomeration of MWCNTs is indispensable if the full potential of MWCNT reinforcements is to be realized.

**Figure 8:**
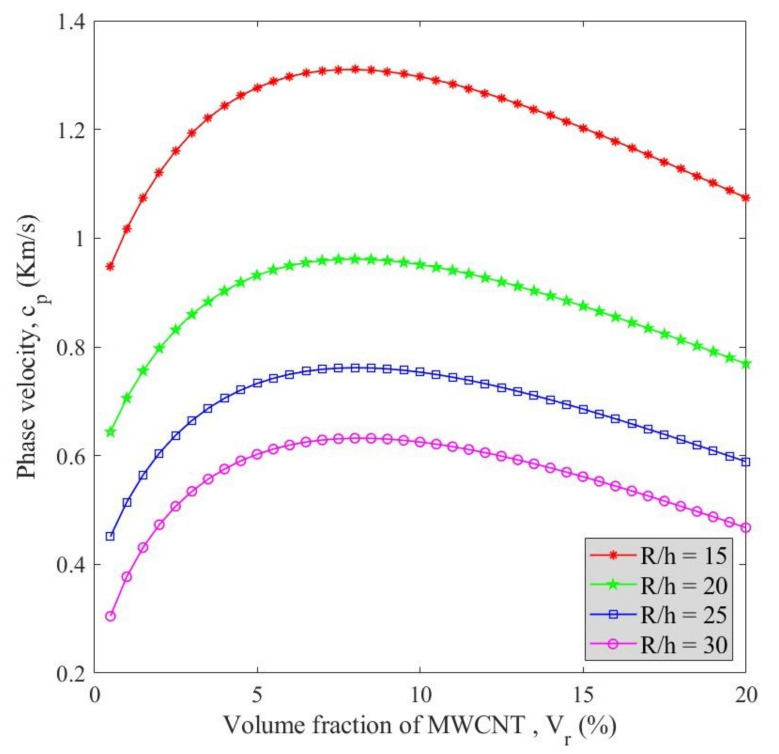
Variation of phase velocity (*c_p_*) versus MWCNTs’ volume fraction (*V_r_*) for different radius to thickness ratios (R/h).

Figure 8 illustrates the variation of phase velocity versus MWCNTs’ volume fraction for different radius to thickness ratios has at *v_x_* = 2000, *V_r_* = 5%, and *β* = 25. As can be seen, this ratio had a decreasing influence such that the phase velocity increased by the decline of the radius to thickness ratio. In other words, the phase velocity is reversely proportional to the radius to thickness ratio. The softening effect caused by the increment of radius to thickness ratio can explain this behavior. At a constant radius to thickness ratio, an enhancement in the MWCNT content raised the phase velocity to its maximum value followed by a gradual decrease which can be assigned to the agglomeration factor. 

**Figure 9:**
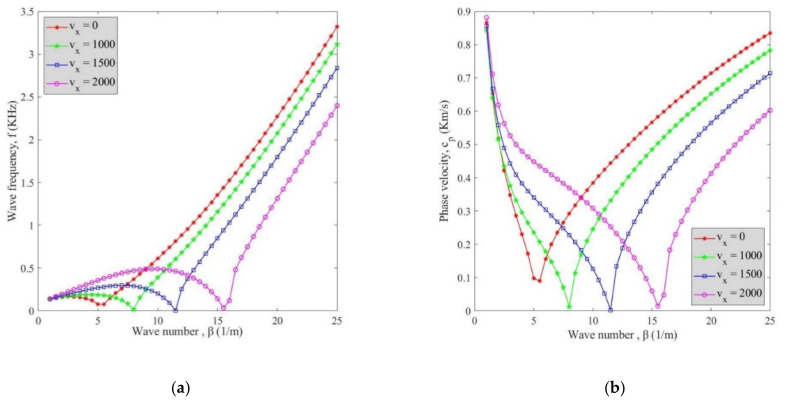
The effect of fluid flow velocities on changes of (**a)** wave frequency and (**b**) phase velocity versus wave number.

Finally, Figure 9 reveals changes of (**a**) wave frequency and (**b**) phase velocity versus the wave number for different fluid flow velocities at *V_r_* = 5% considering all factors. According to diagrams, the cylindrical shell without fluid flow exhibited greater phase velocity and wave frequency as compared to its peers possessing various fluid flow. This means that fluid flow had a reducing influence on changes of phase velocity and wave frequency. The damping wave number also varied by changing the fluid flow velocity. On the other hand, the critical flow velocity can be defined for wave numbers whose natural frequency reached its minimum values. 

## 5. Conclusions 

The present investigation was aimed to assess the wave dispersion in a fluid-conveying MWCNTs-strengthened nanocomposite cylindrical shell considering the influences of the nanofillers’ waviness, agglomeration, and orientation. The effective mechanical properties of nanocomposite cylindrical shells were estimated using a combination of a new form of Halpin-Tsai homogenization model and rule of mixture. Based on this model, the noted effects were investigated implicitly. Viscous fluid flow was considered Newtonian, axially symmetric, laminar, and fully developed. Navier-Stokes equation was applied to study the effect of flow velocity. To this end, FSDT and Hamilton’s principle were used to derive the governing equations of MWCNTs-reinforced nanocomposite cylindrical shell. Eventually, the obtained governing equations were analytically solved and wave frequency and phase velocity values were calculated. To verify present method, the obtained results were compared to other investigations. The most remarkable highlights can be expressed as:

1. The phase velocity and wave frequency of the fluid-conveying MWCNT-reinforced nanocomposite cylindrical shell decreased with enhancing the fluid flow velocity.
2. An increment in the radius to thickness ratio declined the phase velocity and wave frequency of the fluid-conveying MWCNT-reinforced nanocomposite cylindrical shells.
3. Agglomeration and waviness factor affected the mechanical behavior of nanocomposite shells in a decreasing manner.
4. Regardless of the agglomeration factor, an enhancement in the MWCNT content augmented the elastic modulus, wave frequency, and phase velocity.
5. The increment of parameter α plays a decreasing role in the variation of phase velocity.
6. Resolving the MWCNTs agglomeration is vital to enhance the mechanical behavior of nanocomposite cylindrical shells.

## Figures and Tables

**Table 1:** The properties of the constituent materials of the MWCNT-reinforced nanocomposite shells.

Mechanical Properties	Polystyrene [22]	MWCNT [48]
*E* (GPa)	1.9	800
*ρ* (Kg/m^3^)	1050	2100
υ	0.34	0.28

**Table 2:** Comparison of non-dimensional natural frequencies ($\bar{\omega }=R\omega \sqrt{\frac{\rho \left(1-\upsilon^{2}\right)}{E}}$ ) of the cylindrical shell for the C-C boundary condition.

n	Pradhan, Loy, Lam and Reddy [51]	Error (%)	Wang and Wu [52]	Error (%)	Li, Pang, Chen and Du [53]	Error (%)	Present
1	0.0342	2.632	0.0340	3.235	0.0332	5.723	0.0351
2	0.0119	2.521	0.0119	2.521	0.0117	4.274	0.0122
3	0.0072	0	0.0072	0	0.0071	1.389	0.0072
4	0.0089	0	0.0090	1.124	0.0090	1.124	0.0089
5	0.0136	0.735	0.0137	1.460	0.0137	1.460	0.0135

## Appendix A

$$\begin{array}{cc} \; & K_{11}=-A_{11}k_{x}^{2}-\frac{A_{66}}{R^{2}}k_{n}^{2}\\ \; & K_{12}=K_{21}=-\left(\frac{A_{12}+A_{66}}{R}\right)k_{x}k_{n}\\ \; & K_{13}=-K_{31}=i\frac{A_{12}}{R}k_{x}\\ \; & K_{14}=K_{41}=-B_{11}k_{x}^{2}-\frac{B_{66}}{R^{2}}k_{n}^{2}\\ \; & K_{15}=K_{51}=K_{24}=K_{42}=-\left(\frac{B_{12}+B_{66}}{R}\right)k_{x}k_{n}\\ \; & K_{22}=-A_{66}k_{x}^{2}-\frac{A_{11}}{R^{2}}k_{n}^{2}-\frac{A_{55}^{s}}{R^{2}}\\ \; & K_{23}=-K_{32}=i\left(\frac{A_{11}+A_{55}^{s}}{R^{2}}\right)k_{n}\\ \; & K_{25}=K_{52}=-B_{66}k_{x}^{2}-\frac{B_{11}}{R^{2}}k_{n}^{2}-\frac{A_{55}^{s}}{R}\\ \; & K_{33}=-A_{55}^{s}{k_{x}}^{2}-\frac{A_{11}}{R^{2}}+k_{n}^{2}\frac{A_{55}^{s}}{R^{2}}+\rho_{f}h_{f}v_{x}^{2}k_{x}^{2}-\frac{i\mu_{f}h_{f}v_{x}k_{x}}{R^{2}}\left(2+k_{n}^{2}\right)-i\mu_{f}h_{f}v_{x}k_{x}^{2}\\ \; & K_{34}=-K_{43}=i\left(A_{55}^{s}-\frac{B_{12}}{R}\right)k_{x}\\ \; & K_{35}=-K_{53}=i\left(\frac{A_{55}^{s}}{R}-\frac{B_{11}}{R^{2}}\right)k_{n}\\ \; & K_{44}=-D_{11}k_{x}^{2}-k_{n}^{2}\frac{D_{66}}{R^{2}}-A_{55}^{s}\\ \; & K_{45}=K_{54}=-k_{x}k_{n}\left(\frac{D_{12}+D_{66}}{R}\right)\\ \; & K_{55}=-D_{66}k_{x}^{2}-k_{n}^{2}\frac{D_{11}}{R^{2}}-A_{55}^{s}\\ \; & C_{3,3}=2k_{x}\left(\rho_{f}h_{f}v_{x}+\frac{\mu_{f}h_{f}}{R^{2}}\right)+\mu_{f}h_{f}k_{x}^{2}+\frac{\mu_{f}h_{f}}{R^{2}}k_{n}\\ \; & M_{1,1}=M_{2,2}=I_{0}\\ \; & M_{1,4}=M_{2,5}=M_{4,1}=M_{5,2}=I_{1}\\ \; & M_{3,3}=I_{0}+\rho_{f}h_{f}\\ \; & M_{4,4}=M_{5,5}=I_{2} \end{array}$$
